# Benign External Hydrocephalus in a Subgroup of Autistic Children Prior to Autism Diagnosis

**DOI:** 10.1002/aur.70104

**Published:** 2025-08-08

**Authors:** Gal Ben‐Arie, Ilan Shelef, Gal Meiri, Idan Menashe, Ilan Dinstein, Ayelet Arazi

**Affiliations:** ^1^ Radiology Department Soroka University Medical Center Beer Sheva Israel; ^2^ Psychiatry Department Soroka University Medical Center Beer Sheva Israel; ^3^ Azrieli National Centre for Autism and Neurodevelopment Research Ben Gurion University of the Negev Beer Sheva Israel; ^4^ Public Health Department Ben Gurion University of the Negev Beer Sheva Israel; ^5^ Psychology Department Ben Gurion University of the Negev Beer Sheva Israel; ^6^ Cognitive and Brain Sciences Department Ben Gurion University of the Negev Beer Sheva Israel; ^7^ Department of Neurophysiology and Pathophysiology University Medical Center Hamburg‐Eppendorf Hamburg Germany

**Keywords:** autism spectrum disorder, benign external hydrocephalus, CSF, extra‐axial cerebrospinal fluid, MRI

## Abstract

Benign external hydrocephalus (BEH) is evident in < 0.6% of births. It is defined by abnormally large cerebrospinal fluid (CSF) volumes in the subarachnoid space (SAS) and otherwise normal neuroimaging findings before 2 years of age. BEH has not been associated with specific developmental disorders and is not treated because it usually resolves spontaneously. However, quantitative MRI studies have reported that some toddlers with autism exhibit enlarged extra‐axial CSF (EA‐CSF) volumes. Our objective was to determine whether a subgroup of children with autism exhibits both qualitative BEH and quantitative EA‐CSF volume enlargements. We analyzed clinical brain MRI scans in a retrospective sample of 136 children, 5–99 months old, 83 with autism, who were assessed for BEH by neuroradiologists. EA‐CSF volume and total cerebral volume (TCV) were quantified in T2‐weighted scans by manual labeling. Measures were compared across groups while stratifying participants by age. Neuroradiologists reported BEH findings in 33% of autistic children scanned before the age of 2 years old (i.e., before autism diagnosis). Quantitative MRI analyses demonstrated that autistic children in this age group exhibited significantly larger EA‐CSF volumes relative to controls (*t*_(49)_ = 2.89, *p* = 0.006, Cohen's *d* = 0.82) with 30% of autistic children and 9.5% of the controls exhibiting EA‐CSF/TCV ratios > 0.14, a previously suggested threshold of potential clinical relevance. EA‐CSF differences were not apparent in older children. The prevalence of BEH associated with quantifiable EA‐CSF enlargements was remarkably high in toddlers who later developed autism, suggesting a specific autism etiology involving early transient CSF circulation problems with potentially long‐lasting neurodevelopmental impact.

**Summary**

- Benign external hydrocephalus (BEH) is a diagnosis given by radiologists to children who complete MRI scans and exhibit abnormally large cerebrospinal fluid (CSF) volumes.
- CSF is a special fluid that circulates around and within the brain, nourishing brain cells and clearing waste.
- The current study demonstrates that the prevalence of BEH is remarkably high in toddlers under the age of 2 years old who later develop autism.
- The results suggest that up to 30% of autistic children exhibit a specific autism etiology involving early transient CSF circulation problems with potentially long‐lasting developmental impact.

## 1 Introduction

Autism spectrum disorder (ASD) is a heterogeneous neurodevelopmental disorder characterized by difficulties in social communication and the presence of restricted and repetitive behaviors (American Psychiatric Association 2013). Numerous theories, mostly based on MRI studies, have suggested that children with ASD may exhibit structural brain differences during early development. Examples include early increases in total brain volume (TBV) (Courchesne et al. 2003; Hazlett et al. 2017), abnormal amygdala volumes (Herrington et al. 2017; Schumann et al. 2009), and cerebellar anatomy (Allen and Courchesne 2003), which have all been proposed as potential biomarkers for ASD. However, many findings have not been replicated by studies with larger MRI datasets (Haar et al. 2016; Laidi et al. 2022), suggesting that they may not generalize to the broad ASD population. Such mixed findings are common in ASD research and have led to proposals to abandon the search for a single ASD biomarker (Happé et al. 2006). Instead, it may be more useful to search for stratification biomarkers that can identify ASD subgroups with distinct physiology and etiologies (Lombardo et al. 2019).

One such biomarker may be increased volumes of extra‐axial cerebrospinal fluid (EA‐CSF) surrounding the cerebral cortex (Shen 2018). CSF plays a critical role in brain development (Lun et al. 2015), ensuring the delivery of growth factors and signaling molecules (Fame and Lehtinen 2020) while clearing waste and toxins (Ahn et al. 2019; Jessen et al. 2015). Several studies have reported that a sub‐group of young children with ASD exhibit increased EA‐CSF volumes before the age of 4 years old (Shen et al. 2013, 2017, 2018), suggesting that abnormalities in CSF circulation and/or drainage may be associated with ASD development in these children (Shen 2018). More specifically, one study reported that 21 of 159 2–4‐year‐old children with ASD (i.e., 13% of their sample) exhibited an EA‐CSF/total cerebral volume (TCV) ratio higher than 0.14 (Shen et al. 2018), suggesting a biomarker with potential clinical value for identifying ASD children with this etiology. Two large follow‐up studies with ASD children 4 years old and older did not find any differences in EA‐CSF volumes relative to controls (Peterson et al. 2021, 2023), suggesting that EA‐CSF enlargements are transient with differences resolving later in childhood.

It is unknown whether high EA‐CSF/TCV ratios may correspond to a rare radiological diagnosis of benign external hydrocephalus (BEH), which is given to children with an abnormally large subarachnoid space (SAS) above the frontal lobes, a widened frontal inter‐hemispheric fissure, and otherwise normal neuroimaging findings (Zahl et al. 2011). Estimates of BEH prevalence in the general population range from 0.04% (Wiig et al. 2017; Zahl, Egge, Helseth, and Wester 2019) to 0.6% (Gupta and Belay 2008) of live births. BEH is four times more common in boys than girls (as is ASD) and is usually not treated because SAS volumes return to normal spontaneously without treatment (Zahl et al. 2011). To date, BEH has mostly been associated with transient motor and language delays that resolve at later ages, suggesting mild impact on long‐term development (Alvarez et al. 1986; Hellbusch 2007; Yew et al. 2011) with long‐term developmental problems evident mostly in more severe cases that included surgical treatment with a shunt (Zahl, Egge, Helseth, Skarbø, et al. 2019).

In the current study, we assessed the prevalence of BEH findings in a cohort of ASD children and examined their correspondence with EA‐CSF volume enlargements. Unlike previous studies examining EA‐CSF in prospective research samples, here we performed a retrospective study of a clinical sample with ASD and typically developing (TD) children who had been referred to brain MRI scans for similar clinical reasons, including seizures, torticollis, strabismus, deafness, hypotonia, dysmorphism, and other clinical concerns, excluding macrocephaly (Table 2). We hypothesized that if BEH and excessive EA‐CSF volumes are truly indicative of a specific ASD etiology, they would be evident in a substantial subgroup of the examined ASD cohort and not in controls.

## 2 Methods and Materials

### 2.1 Participants

MRI scans of 102 ASD children and 53 TD children were retrospectively extracted from the Soroka University Medical Center (SUMC) patient records (Table 1). ASD participants included all children who had been diagnosed with ASD at SUMC between 2013 and 2020 and whose data were available through the National Autism Database of Israel (Dinstein et al. 2020; Meiri et al. 2017). Of the 731 ASD children in the database at the time, 102 children (~14%) were referred to a brain MRI scan at SUMC between 2011 and 2020. SUMC is the main clinical center where children insured by the Clalit HMO (who cover 70% of the population in southern Israel) can receive an ASD diagnosis and/or a brain MRI scan, thereby yielding a representative community sample of this geographical area.

**TABLE 1:** Characteristics of ASD and TD children in the final sample.

	ASD (mean ± SD)	TD (mean ± SD)
*N*	83	53
Age (months)	36.6 ± 21.5 range: [5, 99]	36.4 ± 22.4 range: [8, 99]
0–24 months old group	17.8 ± 5.96 (*N* = 30) range: [5, 24]	17.8 ± 5 (*N* = 21) range: [8, 23]
25–48 months old group	35.8 ± 7.47 (*N* = 36) range: [25, 47]	36.2 ± 8.1 (*N* = 21) range: [25, 48]
> 48 months old group	71.2 ± 15.5 (*N* = 17) range: [57, 99]	72.5 ± 16.4 (*N* = 11) range: [53, 99]
Age of ASD diagnosis (months)	37.7 ± 18.1	N/A
Sex	Females: 24 (29%)	Females: 16 (30%)
ADOS‐2 (*N* = 60, 72%):		
Total score	18.3 ± 6.9	N/A
SA	14.8 ± 6.3	N/A
RRB	3.5 ± 1.8	N/A
Calibrated severity score (CSS)	7.6 ± 2.7	N/A
Cognitive score (*N* = 39, 46%)		
Total score	66.9 ± 12.3	N/A

ASD children were referred to MRI scans for different reasons (Table 2) before or after their ASD diagnosis. Twenty‐one ASD children were excluded from analyses due to missing or poor‐quality scans (*n* = 16) and due to diagnosis of microcephaly or macrocephaly (*n* = 3), yielding a final ASD sample of 83 children. Excluding those diagnosed with microcephaly or macrocephaly ensured that the measurements of CSF and TCV were not affected by abnormal skull size.

**TABLE 2:** Reasons for referral of ASD and TD children to brain MRI.

ASD	TD
Dysmorphism Strabismus Hypotonia Seizures Deafness Torticollis Suspected genetic disorder (including: X‐ALD, CHARGE syndrome, Fragile X) Suspected metabolic disease Growth hormone deficiency Premature puberty Subarachnoid cyst Setting‐sun eye phenomenon Abnormal mass above the eye Ocular albinism Preterm birth Autism spectrum disorder Developmental delay (including language, motor, and global delay)	Dysmorphism Strabismus Hypotonia Seizures Deafness Torticollis Suspected genetic disorder (including X‐ALD, Neurofibromatosis) Hearing disability Suspected endocrine syndrome Headaches Sleep apnea pilonidal sinus eye ptosis Suspected brain lesion per CT Papilledema Nasal bridge lesion Hypophysis abnormalities Spastic paraplegia

TD children were selected to match the age range of the ASD children (i.e., 5–99 months old). All TD children were referred to MRI for similar reasons (Table 2) and selected if their brain MRI scans showed no radiological findings and their clinical follow‐up outcomes were normal (i.e., no evidence of developmental delays or any clinical neurological condition). These inclusion criteria were intended to yield a sample of MRI scans from TD children with minimal developmental concerns who were referred to MRI for mild clinical concerns that were later eliminated, thereby creating a sample that would be closest to that expected of TD children in the general population.

### 2.2 ASD Diagnosis

As required by the Israeli health ministry, all ASD children were diagnosed by both a physician (child neurologist or psychiatrist) and a developmental psychologist, according to DSM‐5 criteria (American Psychiatric Association 2013) and best clinical judgment. Of the 83 children with ASD included in the final analysis, 60 children completed the Autism Diagnostic Observation Schedule, 2nd edition (ADOS‐2) (Lord et al. 2012), which was performed by a clinician with research reliability (Table 1). Of these children, 32 completed the ADOS‐2 assessment before the MRI scan (mean gap: 11 ± 14 months, range: [0.3, 48] months) and 28 after the MRI scan (mean gap: 23 ± 20 months, range: [1, 88] months). In addition, 39 of the ASD children completed a cognitive assessment using the Bayley Scales of Infant Development (Bayley 2006) or the Wechsler Preschool and Primary Scale of Intelligence (Wechsler 2002), which were performed by a licensed developmental psychologist together with the ADOS‐2 (Table 1).

### 2.3 MRI Acquisition

MRI scanning was performed with a 1.5T Achieva or 3.0T Ingenia Philips MRI scanner (Philips Medical Systems, Best, The Netherlands). The 1.5T Achieva system was equipped with a six‐channel head coil. T2‐weighted (T2w) scans were performed with a turbo spin‐echo sequence with TE/TR = 5730/110 ms, a field‐of‐view of 210 mm, slice width of 4.5 mm, in‐plane resolution of 0.56 × 0.69 mm, and a flip angle of 90°. The 3T Ingenia system was equipped with a 15‐channel head coil. T2w scans were performed with a multi‐vane turbo spin‐echo with TR/TE of 4000/108 ms, SPIR fat suppression, slice thickness/gap of 3.8/1.0 mm, and a field‐of‐view of 230 mm. All children in both groups were sedated during the MRI scans. All scans were interpreted by a pediatric neuroradiologist with over 15 years of experience (author I.S.). BEH diagnosis was noted when the SAS above the frontal lobes was enlarged, the frontal inter‐hemispheric fissure was widened, and there were no other major neuroimaging findings.

### 2.4 MRI Analyses

**FIGURE 1:**
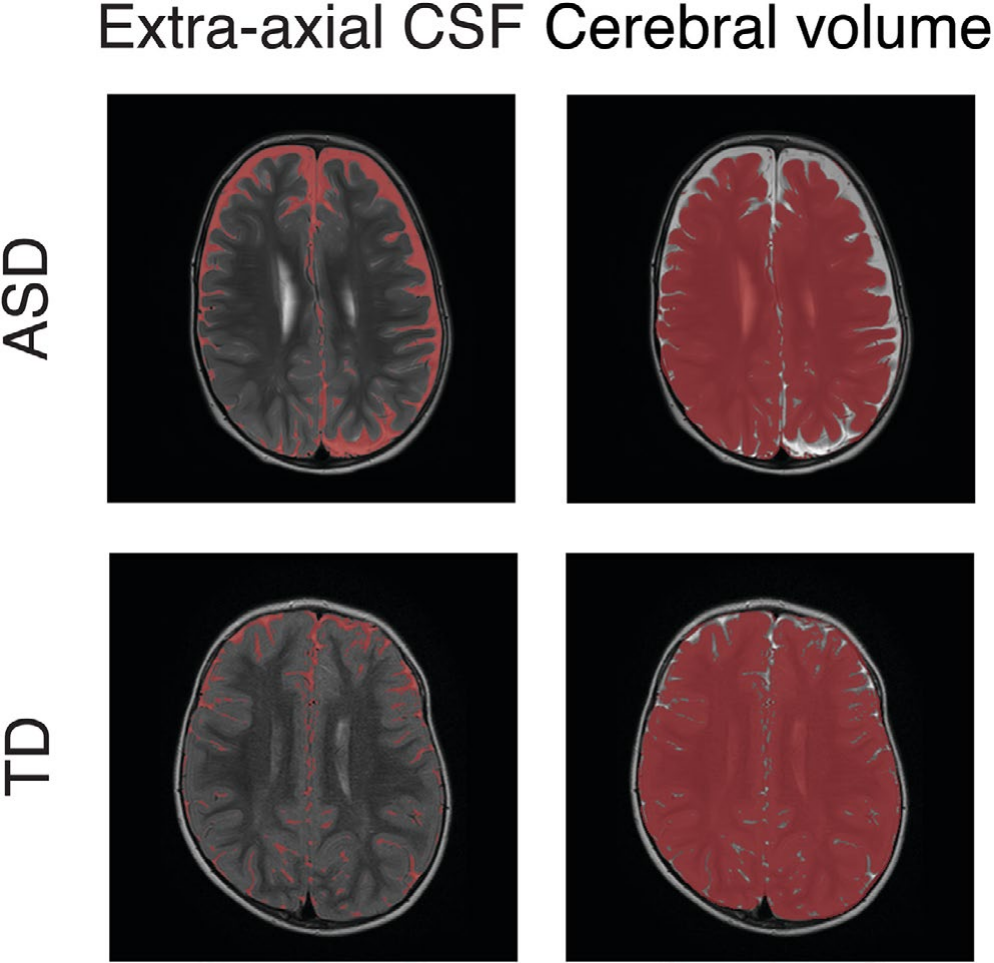
EA‐CSF and TCV labeling. Examples of T2‐weighted images with segmentation of EA‐CSF volumes (left) and TCV (right) of an ASD child (top) and a TD child (bottom), both scanned at the age of 26 months.

EA‐CSF was manually identified on the T2‐weighted scans (Figure 1) in a semi‐automated manner by three annotators (A.A and two second year radiology residents) who were blind to the child's diagnosis. First, a 3D rectangle was manually marked across slices in each of the lateral ventricles. The mean image intensity of the selected voxels was computed, and 50% of this value was set as a CSF detection threshold. Second, we selected all voxels with values above the threshold as CSF (in T2w scans, voxels containing CSF have high image intensity values). This procedure was performed repeatedly while altering the threshold and visually inspecting the scans for each participant separately such that the average threshold was set to 54.1% (±6.3%). After selecting the optimal threshold per participant, annotators used the itk‐SNAP software (www.itksnap.org) to visually inspect the CSF labeling and manually correct it. Ventricles were manually removed, and only EA‐CSF voxels above the anterior‐posterior commissure horizontal plane were included in the final analysis, as performed by previous studies (Peterson et al. 2021, 2023; Shen et al. 2013, 2017). TBV, excluding the brain stem and cerebellum, was manually annotated by two radiology residents who were blind to the children's diagnoses using the itk‐SNAP software. TCV was calculated by subtracting CSF voxels from the voxels marked as TBV (Figure 1).

### 2.5 Statistical Analysis

All statistical analyses were performed using MATLAB (Mathworks Inc. United States). EA‐CSF volume, TCV, and their ratio were compared across ASD and TD groups using linear regression models with diagnosis, age, sex, and scanner type (1.5T or 3T) as predictors. Relationships between volumes and age were quantified using Pearson's correlation coefficients. We also performed a four‐way ANOVA analysis with diagnosis, age group, sex, and scanner type as main factors. Three age groups were defined with children who were 0–24 months old (30 ASD, 21 control), 24–48 months old (36 ASD, 21 control), or 49–99 months old (17 ASD, 11 control). Post hoc comparisons between ASD and TD children per age group were performed using two‐tailed *t*‐tests when the initial ANOVA results indicated significant differences. We used Bonferroni correction to control for multiple comparisons across the three age groups.

## 3 Results

**FIGURE 2:**
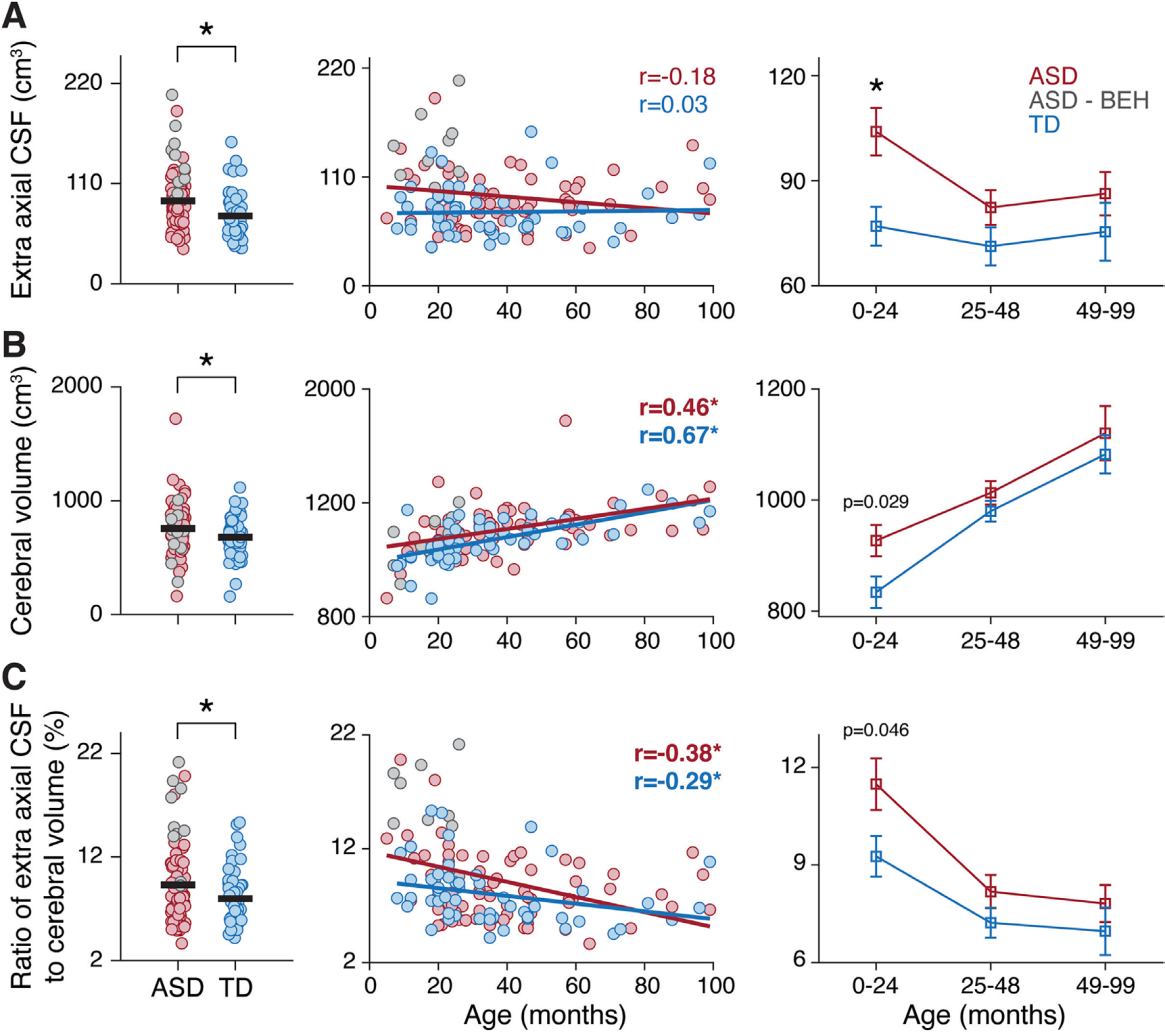
Comparison of EA‐CSF volume (A), TCV (B), and their ratio (C) across ASD and TD children. Scatter plots present individual ASD children with (gray) and without (red) BEH findings as well as TD children (blue). Left panels: Comparison across groups with children of all ages. Each circle represents a single child, and the black lines represent group means. Asterisks: significant difference, four‐way ANOVA analysis, *p* < 0.05. Middle panels: Scatter plot of each measure and its relationship with age. Solid lines: least squares linear fit. Pearson's correlation coefficients are noted. Asterisks: significant correlation, *p* < 0.05. Right panels: Comparison of ASD and TD children, separately for each of the three age groups. Asterisks: significant difference, two‐tailed *t*‐test, *p* < 0.0167 (Bonferroni corrected).

Linear regression analyses revealed that diagnosis (ASD/TD) and scanner type were significant predictors of EA‐CSF; diagnosis, age, and sex were significant predictors of TCV volumes; and diagnosis, age, and scanner type were significant predictors of EA‐CSF/TCV ratios (Table 3). The significant relationship between EA‐CSF/TCV ratios and age of the children motivated us to split them into three age groups (0–24, 25–48, and > 48 months‐old; Table 1) when performing all further analyses. Note that the clinical definition of BEH is specific to infants and toddlers younger than 24 months of age (Zahl et al. 2011) and further motivates separate analysis of children in the selected age groups. To demonstrate differences across diagnostic groups and relationship with age, we present scatter plots of the three variables (Figure 2).

**TABLE 3:** Results of linear regression models explaining EA‐CSF volumes, TCV, and EA‐CSF/TCV ratios.

Predictor	*t*‐value	*p*
EA‐CSF		
Diagnosis	3.14	0.002*
Age	−0.98	0.33
Sex	−0.2	0.8
Scanner	−2.83	0.005*
TCV		
Diagnosis	2.55	0.01*
Age	7.2	< 0.0001*
Sex	−2.6	0.01*
Scanner	1.24	0.21
EA‐CSF/TCV ratio		
Diagnosis	2.38	0.02*
Age	−3.91	0.0001*
Sex	0.71	0.48
Scanner	−3.48	0.0007*

*Note*: Significant predictors are marked with an asterisk.

A four‐way ANOVA analysis with diagnosis, age group, sex, and scanner type as main factors revealed that EA‐CSF differed significantly across ASD and TD children ($F_{\left(1,110\right)}$ = 9.31, *p* = 0.003) and across the three age groups ($F_{\left(2,110\right)}$ = 3.23, *p* = 0.04), but not across sex groups or scanners ($F_{\left(1,110\right)}$ < 0.35, *p* > 0.56). TCV differed significantly across ASD and TD children ($F_{\left(1,110\right)}$ = 5.35, *p* = 0.02), age groups ($F_{\left(2,110\right)}$ = 10.23, *p* = 0.0001), and sex ($F_{\left(1,110\right)}$ = 4.92, *p* = 0.03), but not across scanners ($F_{\left(1,110\right)}$ = 2.37, *p* = 0.12). Finally, EA‐CSF/TCV ratios differed significantly across ASD and TD children ($F_{\left(1,110\right)}$ = 4.96, *p* = 0.03) and age groups ($F_{\left(2,110\right)}$ = 8.89, *p* = 0.0003), but not across sex or scanners ($F_{\left(1,110\right)}$ < 1.41, *p* > 0.24).

Post hoc analyses performed separately for each age group (Figure 2) revealed consistent differences across ASD and control groups only in the youngest age group. Significantly larger EA‐CSF in the ASD group was apparent only in the youngest age group (*t*_(49)_ = 2.89, *p* = 0.006, Cohen's *d* = 0.82) and not in the older age groups (age 24–48: *t*_(54)_ = 1.44, *p* = 0.15, Cohen's *d* = 0.4; age 49–99: *t*_(26)_ = 1.07, *p* = 0.29, Cohen's *d* = 0.42). TCV was also larger in the ASD group in the youngest age group (*t*_(49)_ = 2.26, *p* = 0.029, Cohen's *d* = 0.64) and not in older age groups (age 24–48: *t*_(54)_ = 1.1, *p* = 0.29, Cohen's *d* = 0.29; age 49–99: *t*_(26)_ = 0.57, *p* = 0.58, Cohen's *d* = 0.22). EA‐CSF/TCV ratios were larger in the ASD group in the youngest age group (*t*_(49)_ = 2.05, *p* = 0.046, Cohen's *d* = 0.58) and not in the older age groups (age 24–48: *t*_(54)_ = 1.26, *p* = 0.21, Cohen's *d* = 0.35; age 49–99: *t*_(26)_ = 0.9, *p* = 0.36, Cohen's *d* = 0.36). Note that the TCV and EA‐CSF/TCV ratio analyses do not survive Bonferroni correction for multiple comparisons. Nevertheless, these findings demonstrate an increase in EA‐CSF volumes that is above and beyond observed increases in TCV in ASD children younger than 2 years old.

### 3.1 BEH in the ASD Group

Of the 83 ASD children examined in this study, 10 children had BEH findings noted in their radiological examinations (gray circles, Figure 2). All these children were in the youngest age group (i.e., under 2 years old) and eight of the 10 children had an EA‐CSF/TCV ratio > 0.14, suggesting a strong relationship between this qualitative radiological finding and the corresponding quantitative measure.

### 3.2 Re‐Analysis While Excluding ASD Children With Major Radiological Findings

ASD and TD children examined in the current study were referred to a clinical MRI scan to assess potential pathologies in brain development. While the included TD children did not have any major radiological findings, some of the ASD children did. We, therefore, performed a second analysis while excluding 15 ASD children who had radiological findings including cortical dysplasia, periventricular leukomalacia, mesial temporal sclerosis, congenital Cytomegalovirus, and posterior fossa cyst that may be expected to impact brain parenchymal or CSF volumes.

**FIGURE 3:**
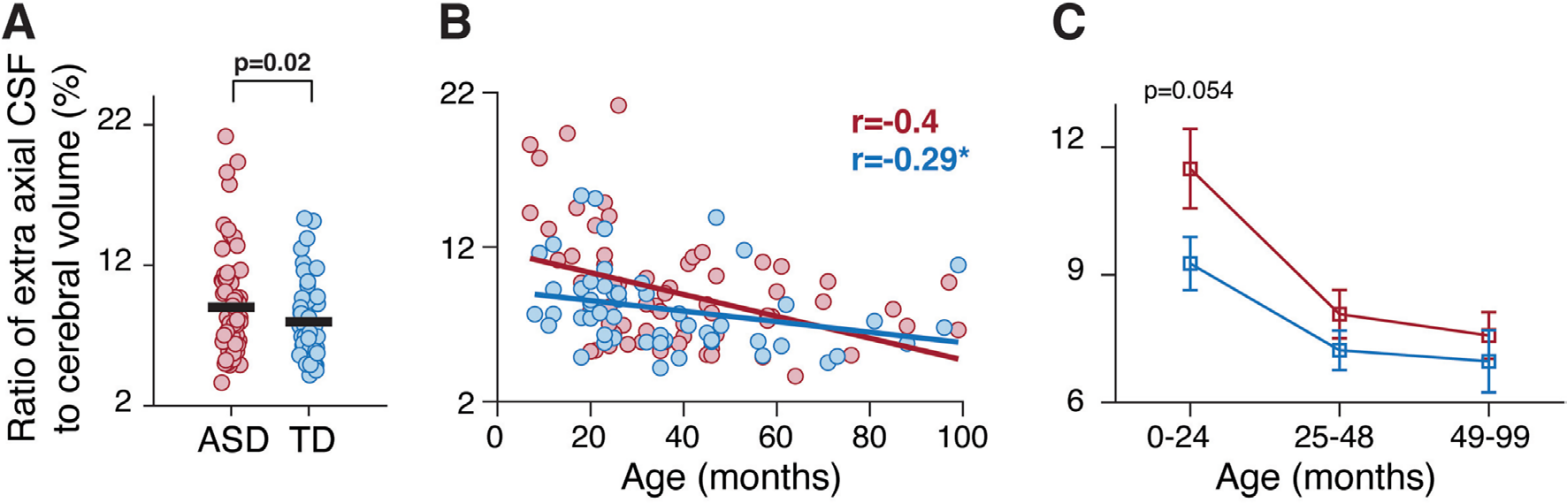
Comparison of EA‐CSF/TCV ratios across ASD (red) and TD (blue) children after excluding 15 children with radiological findings. (A) Comparison across groups (all children). Each circle represents a single child, and the black lines represent group means. (B) Scatter plot of EA‐CSF/TCV ratios and their relationship with age. Solid lines: least squares linear fit. Pearson's correlation coefficients are noted. Asterisks: significant correlation, *p* < 0.05. (C) Comparison across ASD and TD groups, separately for each of the three age groups.

This analysis, performed with 68 ASD and 53 TD children, reproduced similar results. A four‐way ANOVA analysis with diagnosis, age group, sex, and scanner type as main factors revealed that EA‐CSF differed significantly across ASD and control children ($F_{\left(1,97\right)}$ = 5.52, *p* = 0.02) and across the three age groups ($F_{\left(2,97\right)}$ = 11.8, *p* < 0.0001), but not across sex groups or scanners ($F_{\left(1,97\right)}$ < 2.43, *p* > 0.12). Two‐tailed *t*‐tests performed separately for each age group revealed a marginally significant increase of EA‐CSF/TCV ratio in the ASD group only in the youngest age group (*t*_(40)_ = 1.98, *p* = 0.054, Cohen's *d* = 0.61, Figure 3C).

### 3.3 Subgroup of Young ASD Children With BEH and Large EA‐CSF Volumes

The group differences described above in children under the age of 2 years old were driven by a subset of children who had extreme EA‐CSF volumes. Previous studies have suggested that ASD children with an EA‐CSF/TCV ratio ≥ 0.14 may represent a subgroup with unique pathophysiology (Shen et al. 2018). In our study, nine of 30 ASD children who were younger than 24 months (i.e., 30% of this age group) exhibited a ratio ≥ 0.14, in contrast to two of the 21 TD children in this age group (~9.5%). Note that 5 of 30 ASD children (i.e., 16.6%) and no TD children had EA‐CSF/TCV ratios ≥ 0.16, suggesting that a slightly higher threshold may yield 100% specificity.

### 3.4 Increased EA‐CSF May Be Weakly Associated With the Severity of ASD Symptoms

We estimated the relationship between the severity of autism symptoms, as assessed using the ADOS‐2 calibrated severity scores (CSS, see Section 2), and each of the three anatomical measures. Of the 83 children included in the main analyses, 60 children completed the ADOS‐2 assessment. We found marginally significant correlations between their ADOS‐2 CSS scores and EA‐CSF volumes (*r*_(60)_ = 0.23, *p* = 0.07) as well as EA‐CSF/TCV ratios (*r*_(60)_ = 0.23, *p* = 0.08), but not with TCV (*r*_(60)_ = −0,04, *p* = 0.77). Isolating the 23 children who completed ADOS‐2 assessments and were scanned before the age of 24 months did not reveal any significant correlations for this subgroup (*r*_(23)_ < 0.16, *p* > 0.46).

## 4 Discussion

The results revealed that a large subgroup of children with ASD (8 of 30; ~26%) who were scanned before the age of 2 years old exhibited a combination of BEH findings and EA‐CSF/TCV ratios exceeding 0.14. This is remarkable given that the estimated prevalence of BEH in the general population ranges from 0.04% (Wiig et al. 2017; Zahl, Egge, Helseth, and Wester 2019) to 0.6% (Gupta and Belay 2008). While BEH findings are defined qualitatively (Zahl et al. 2011), our results demonstrate that they correspond well with quantitative measures of EA‐CSF (Figure 2). This suggests that BEH and EA‐CSF/TCV ratios may represent a novel biomarker indicative of early CSF circulation abnormalities that may play a role in the ASD etiology of a specific subgroup of children (Shen 2018).

The findings of this study support accumulating evidence demonstrating that excessive EA‐CSF volumes during early development characterize a potentially unique sub‐group of ASD children. In the current study, large EA‐CSF/TCV ratios exceeding 0.14 were apparent in 30% of ASD children scanned before the age of 2 years old (Figure 2). This strengthens findings from a recent prospective study where ~13.2% of 3‐year‐old ASD children exhibited ratios above this value (Shen et al. 2018). Moreover, two previous studies reported that early EA‐CSF volumes at 6 and 24 months of age were significantly larger in high‐risk baby siblings of ASD children who also developed ASD versus those who did not (Shen et al. 2013, 2017). Hence, similar findings were apparent in multiple previous prospective studies performed in a research university setting and in the current retrospective study of children scanned for clinical reasons in a medical center setting.

Note that in the current study, applying a slightly higher EA‐CSF/TCV threshold of 0.16, as opposed to 0.14 proposed previously (Shen et al. 2018), was able to identify 16.6% of the young ASD sub‐group with 100% specificity (i.e., no TD children had EA‐CSF/TCV values above this threshold). Optimal threshold values are likely to vary across studies with different sample characteristics, differences in the type of analyzed scans (T1w vs. T2w), and differences in the analysis approach (automated vs. manual labeling of EA‐CSF and TCV). Nevertheless, it is remarkable that two studies using such different sampling approaches and analysis techniques converge on very similar thresholds. Further research with large cohorts is highly warranted for establishing optimal clinical thresholds.

ASD children scanned at older ages in the current study (4–8.3 years old) did not exhibit significant EA‐CSF or TCV enlargements relative to controls (Figure 2). Indeed, two large studies have recently reported that EA‐CSF volumes of ASD children above the age of four do not differ significantly from those of controls (Peterson et al. 2021, 2023). Taken together, these findings suggest that clinical BEH findings and corresponding excessive EA‐CSF/TCV ratios in the sub‐group described above are a transient characteristic that appears during early development and resolves later in childhood as the children mature.

### 4.1 ASD Etiology Involving Impaired EA‐CSF Circulation?

Recent studies have revealed that CSF circulation plays many critical roles during early brain development (Fame et al. 2020) including delivery of growth factors and essential metabolites (Fame and Lehtinen 2020) as well as removal of waste (Ahn et al. 2019). Indeed, multiple types of CSF circulation impairments, including congenital and acquired forms of hydrocephalus, can cause severe developmental disorders (Tully and Dobyns 2014; Wright et al. 2016) with underlying mechanisms that are poorly understood (MacAulay 2021). The findings reported in this and previous studies (Shen et al. 2013, 2018) suggest that a milder, transient form of hydrocephalus during early development (BEH) may be associated with later development of ASD.

It is important to note that early insults to neural development during critical periods, which could potentially be caused by CSF circulation problems, often have lasting effects on neural circuit formation and maturation even in cases where the early insult is later resolved. For example, the impact of transient monocular deprivation during the critical period of visual system development causes long‐lasting visual system deficits even if monocular deprivation is resolved (Hubel and Wiesel 1970). Further exploration of the potential mechanisms underlying the relationship between early BEH and later development of ASD is, therefore, highly warranted and may yield targeted interventions for this specific sub‐group of ASD cases.

## 5 Limitations

The current study had several limitations. First, we compared ASD and TD participants who were referred to a brain MRI scan for clinical reasons and may not faithfully represent the ASD and TD general populations. Note, however, that prospective research samples in a university setting also do not necessarily generalize to the broad population due to a variety of common social, racial, and cognitive sampling biases (Lewinn et al. 2017; Russell et al. 2019). Hence, the current sample, with its unique characteristics, extends previous findings from other types of samples. Second, our quantification of EA‐CSF and TCV was based on T2w scans with poorer spatial resolution than previously utilized T1w MRI scans. Our analyses may, therefore, be based on less accurate manual annotation of TCV and EA‐CSF volumes. Third, the manual annotation of EA‐CSF and TCV used in the current study would not be conducive to work with larger datasets where application of automated techniques would be required. While automated techniques have been developed for analyses of T1w scans (Shen et al. 2017), extending these techniques to T2w scans has not been carried out to date. Fourth, the sample size per age group was relatively small, with < 60 children in each subgroup, thereby impacting the statistical power of the current study. Finally, the qualitative definition of BEH suggests that there is likely to be variability in how different neuroradiologists assign this diagnosis to different children. We did not examine inter‐rater reliability across neuroradiologists in the current study.

## 6 Conclusions

Clinical BEH findings corresponding to abnormally large EA‐CSF/TCV ratios represent a potential early biomarker for a specific ASD etiology involving early CSF circulation problems. This etiology seems to appear in 13%–30% of ASD children whether ascertained in prospective university‐based research studies or in retrospective community clinical samples. These findings suggest that toddlers with BEH should be prioritized for early developmental assessments and are likely to be at a higher likelihood of developing ASD. They also suggest that BEH may not be an entirely benign diagnosis and warrant further research to determine its potential neuropathological impact.

## Ethics Statement

The study was approved by the Helsinki Committee at Soroka University Medical Center (approval number SOR‐077‐21).

## Conflicts of Interest

The authors declare no conflicts of interest.

## Data Availability

Total brain volumes and extra‐axial CSF measures, as well as meta‐data will be made available upon publication. Meta‐data includes age, gender, presence of BEH or other radiological findings, as well as ADOS scores and cognitive scores. Structural MRI scans will not be made available to protect children's privacy.
